# Super-Efficient Synthesis of Mesh-like Superhydrophobic Nano-Aluminum/Iron (III) Oxide Energetic Films

**DOI:** 10.3390/ma12020234

**Published:** 2019-01-11

**Authors:** Xiaogang Guo, Taotao Liang

**Affiliations:** 1 Chongqing Key Laboratory of Inorganic Special Functional Materials, College of Chemistry and Chemical Engineering, Yangtze Normal University, Chongqing 408100, China; 2 Faculty of Materials and Energy, Southwest University, Chongqing 400715, China; liangtaotao@email.swu.edu.cn

**Keywords:** aluminum/iron (III) oxide, electrophoretic deposition, perfluorodecyltriethoxysilane, self-cleaning, great stability

## Abstract

In this study, a novel superhydrophobic nano-aluminum/iron (III) oxide composite has been prepared by a facile one-step process of electrophoretic deposition, with wide potential applications. The optimal suspension included ethanol, acetyl-acetone, and the additives of fluorotriphenylsilane and perfluorodecyltriethoxysilane. The microstructure, wettability, and exothermic performance were analyzed by field emission scanning electron microcopy (FESEM), X-ray diffraction (XRD), water contact angle measurements, and the differential scanning calorimetry (DSC) technique. The water contact angle and the heat-release of the target composites could reach to ~170° and 2.67 kJ/g, and could still keep stable, after exposure for six months, showing a great stability. These results provided an exquisite synthesis of ideas, for designing other superhydrophobic energetic materials with self-cleaning properties, for real industrial application.

## 1. Introduction

Nano energetic materials or metastable interstitial composites (MICs), including Al/CuO, Al/Fe_2_O_3_, Al/Bi_2_O_3_, Al/MO_3_, and unconventional substances of Al/AgIO_3_, Al/I_2_O_5_, and Al/KMnO_4_ etc., have attracted steadily growing attentions, due to their higher energy density, faster energy release rates, higher explosion intensity, and more efficient reaction process, resulting from fuller interface contact between the reactants [1,2,3,4,5]. Up to now, based on a great number of advantages, they have been the subject of intense research work for fields of blasting, welding, automotive air-bag propellants, hardware destruction, gas sensor, etc. [6,7,8,9,10]. Notably, the heat-release (Q) of the theoretical stoichiometric Al/Fe_2_O_3_, as a classical thermite system, is more than 900 cal/g, and its adiabatic temperature is up to 3135 K, with wide potential applications [11].

Recently, abundant efforts have been devoted to fabricate Al/Fe_2_O_3_ energetic materials, by using various techniques, including simple physical mixing [12,13], magnetron sputtering [14], arrested reactive milling (ARM) [15], electrospinning [16], vapor deposition [17], sol-gel technique [18], etc. Most relevant research works are mainly concerned with simplifying the synthesis technique, optimizing their morphology, or designing new structures to improve the exothermic capacity or develop the utilization rate of energy. For example, Dadbakhsh and Hao designed an Al/Fe_2_O_3_ powder mixture distributed uniformly, by using selective laser melting [19]. The self-assembly and solvent-based mixing techniques have been used to prepare an Al/Fe_2_O_3_ nanothermite with the Fe_2_O_3_ as nanotubes [20]. In addition, the AP/Al/Fe_2_O_3_ ternary energetic materials have been successfully fabricated by sol-gel, wet impregnation, and solvent anti-solvent processes, by Gao et al. [21]. However, the key components of nano-Al and Fe_2_O_3_ powders in energetic materials are hydrophilia or surperhydrophilia, leading to performance attenuation. Thus, it is rather interesting to develop the exothermic stability and nature environment resistance to develop in energetic materials. The one commonly used method for preserving target energetic materials is by virtue of a nitrogen or argon gas seal bag or equipment. Moreover, Zhou et al. have proposed the glancing angle deposition technique and magnetron sputtering deposition process, to obtain the highly superhydrophobic Mg/Fluorocarbon core/shell nano-energetic arrays, with a static contact angle up to 162° [22]. The Al/CuO, with excellent superhydrophobicity, has been fabricated by chemical vapor deposition with an atomic layer deposition technology, by Collins et al. [23]. However, the most recent reported technologies, generally, are high in cost and complicated. Thus, it is still an impassable bottle-neck, to design novel Al/Fe_2_O_3_ energetic materials with self-protection and high-exothermic capacity.

**Figure 1:**
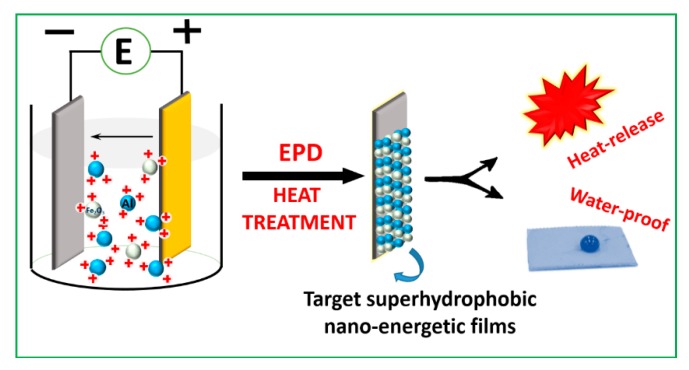
Schematic diagram of the preparation of the superhydrophobic nano-Al/Fe_2_O_3_ films (SAFFs) by a novel one-step process.

The electrophoretic deposition (EPD) method have been reported in the literature, to be a low-cost and highly efficient technique for fabricating promising films or coatings [24,25,26]. As for the Al/Bi_2_O_3_ thermite system, a facile two-step method of EPD and surface modification was introduced, in our previous research work, to construct a superhydrophobic Al/Bi_2_O_3_; their exothermic stability could be maintained for two years, which is of great benefit for practical applications [27]. Moreover, the mentioned method has also been applied to the Al/CuO system [28]. The focus of this work was to attempt to prepare self-protected or superhydrophobic Al/Fe_2_O_3_ materials, by using an improved one-step process of EPD, based on the two-steps technique, to enhance their adaptive capacity in the real, natural environment. The corresponding mechanism diagram is displayed in Figure 1. Additionally, the superhydrophobicity and exothermic stability and water-proof or self-cleaning property of the product composite films have been systematically studied in detail.

## 2. Materials and Methods

### 2.1. Reagents and Materials

Nano-Al (50 nm, 99.9%), nano-Fe_2_O_3_ (30 nm, 99.5%), fluorotriphenylsilane, and perfluorodecyltriethoxysilane were purchased from the Aladdin Industrial Corporation (Shanghai, China), and stored in a vacuum glove box. Acetylacetone and ethanol were purchased from Kelong Industrial Inc., Chengdu, China and were used as received. All other reagents were of analytical grade without further purification.

### 2.2. Preparation of the Superhydrophobic Nano-Al/Fe_2_O_3_ Films (SAFFs)

Commercial aluminum sheet (99%), with an effective electrode area, were utilized as the anode and cathode materials, which were polished by 100^#^–800^#^ grit sandpapers, and were washed by ethanol and deionized water, repeatedly. Then, treated and dried electrodes were used for the following preparation process of the superhydrophobic nano-Al/Fe_2_O_3_ films (SAFFs). In this study, a stable dispersion for EPD was obtained by adding a solid loading of 0.5 g/L with a mole ratio of Al and Fe_2_O_3_ of 2:1 to a mixture of ethanol, acetylacetone, fluorotriphenylsilane, and perfluorodecyltriethoxysilane, with volume ratio of 1:1:10^−2^:10^−2^, and sonicating them for 30 min, in an ultrasonic apparatus (KQ5200DE, Kunshan Ultrasonic Instrument Company, Kunshan, China), with 200 W, to avoid an agglomeration of nanoparticles, as far as possible. The distance of electrodes was controlled at 1 cm, and the field strength during EPD was set as 100 V cm^−1^. After an efficient EPD process (DYY11, Beijing 61 Instrument Factory, Beijing, China), electrophoretic assembly superhydrophobic films were dried, at 373 K, in a vacuum oven (DGG-9076AD, Shanghai Qixin Scientific Instrument Co., Ltd., Shanghai, China), for 30 min, to remove the liquid impurities. At last, the deposited films were treated by microwave heating (WBBX-2, WKTR Science and Technology Ltd., Beijing, China), for 10 min at a certain power, and then stored in a vacuum drying oven (DGG-9076AD, Shanghai Qixin Scientific Instrument Co., Ltd., Shanghai, China), for the performance analysis.

### 2.3. Characterization

The microstructures and materials composition the of SAFFs were characterized by field emission scanning electron microscope (FESEM, JSM-7800F, Tokyo, Japan) and X-ray diffractometer (XRD-6000, Shimadzu, ZD-3AX, Inc., Tokyo, Japan). The wettability was analyzed by an optical contact angle meter (HARKE-SPCA, Beijing, China) and a digital camera (D7000, Nikon, Tokyo, Japan). The following exposure tests were conducted in the open-door real nature environment, for different times. The different humidity levels were controlled by using a salt-spray test chamber (YWX/Q, YSL, Inc., Beijing, China) to explore the stability of the product. The heat output (Q) of the products were analyzed by using a differential scanning calorimetry (DSC, STA449F3, NETZSCH, Selb, Germany) on a freestanding ceramic crucible, with a temperature range of 298 K–1173 K, under a high purity (99.999%) argon environment. 

## 3. Results and Discussion

### 3.1. Characterization of the Product—SAFFs

**Figure 2:**
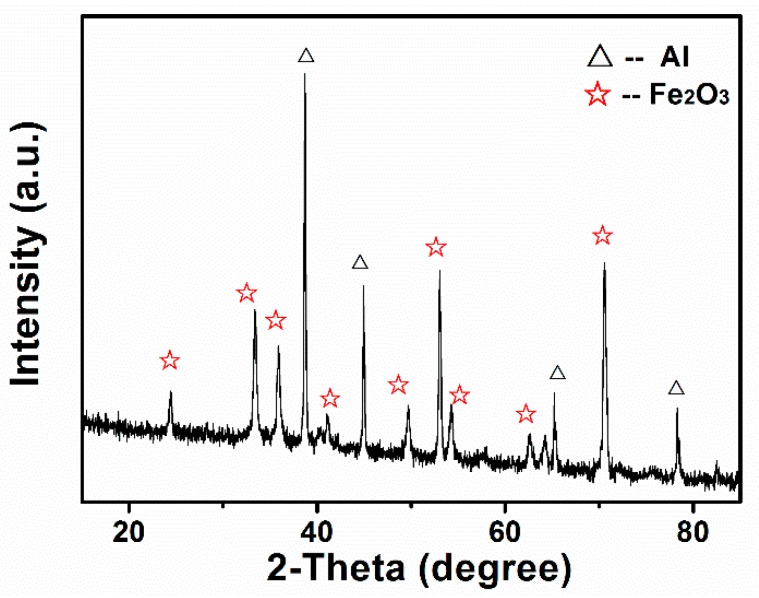
The typical XRD spectra of the SAFFs.

Figure 2 shows the XRD result of the fabricated SAFFs. Clearly, all mainly diffraction lines for the Al (04-0787, the Fm-3m (225)), and the Fe_2_O_3_ (33-0664, R-3c (167)) were identified, demonstrating the presence of Al and Fe_2_O_3_, in the product, which is characteristic of nano-composite films deposited by the EPD technique. In addition, no peak for Al_2_O_3_ or Fe indicated no reaction between the Fe_2_O_3_ and the Al, during a typical EPD process.

**Figure 3:**
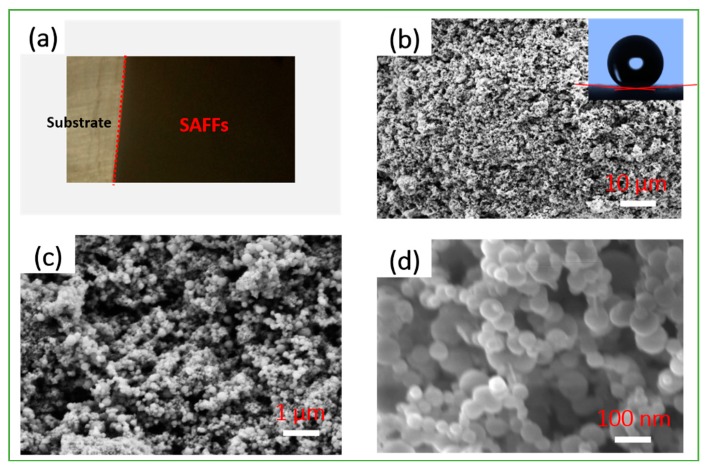
The optical photograph (**a**) and typical SEM images (**b**) of the product. Following are the high resolution FESEM images (**c**,**d**) of the samples. The image embedded in Figure 3b shows the static hydrophobic angle of ca. 170°.

Figure 3 displays the top-view optical and macroscopic SEM images of the target SAFFs. As shown in Figure 3a, the product surface (in the black part) was relatively uniformly-distributed, with no locally macroscopic agglomerate areas, indicating that a suspension including ethanol, acetyl-acetone, and fluorotriphenylsilane and perfluorodecyltriethoxysilane as additives, was the suitable dispersant for this electrophoresis assembly. The higher resolution FESEM image in Figure 3b shows the special mesh-like microstructures in the SAFFs, which provided the structural foundation for improving the superhydrophobicity or weather-proof property, and contributed to the heat-release during the exothermic reaction (Equation (1)).

(1) $$2\mathrm{Al}+{\mathrm{Fe}}_{2}O_{3}\to {\mathrm{Fe}}_{2}O_{3}+2\mathrm{Fe}+\Delta Q$$

Moreover, the component composition (Al and Fe_2_O_3_ particles) of the product were still nano-scale, as can be clearly seen in Figure 3c,d, which were conducive to the largely increasing contact areas among the reactants, and the decreasing mass-transfer length, during the exothermic chemical process [27,29]. 

### 3.2. Wettability

The wettability of the product was systematically analyzed for investigating its hydrophobic performance. A water droplet with a volume of 5 μL on the product surface (it was difficult to do this due to the rather small rolling angle, <1°, as shown in Table 1) was close to a sphere in the typical Cassie state [28,30,31], as seen in the photo embedded in Figure 3b. The corresponding water contact angle was measured at ca. 170°, which meant that the SAFFs were outstandingly superhydrophobic [32,33,34,35]. It is worth mentioning that the samples from the different parallel experiments showed similar results, as seen in Table 1. 

In addition, the water droplet dynamic impact test was used here, to examine the water-proof property of the samples. The whole impact process of a dyed water droplet on the target surface process included the five steps of the initial state (I), the falling process (II), the contact process (III), the seceding state (IV), and the rebounding process (V). Due to the abundant air bubbles captured by the porous structures in the SAFFs [36], the droplet could secede quickly after a rather short contact time, with the superhydrophobic surface, and bounce off, which is demonstrated in Video S1 in the Supplementary Materials. Moreover, when the SAFFs were placed at a small angle, the impact process of the water droplet was also realized at a fast speed, as clearly seen in Video S2 in the Supplementary Materials.

### 3.3. Thermal Analysis

**Figure 4:**
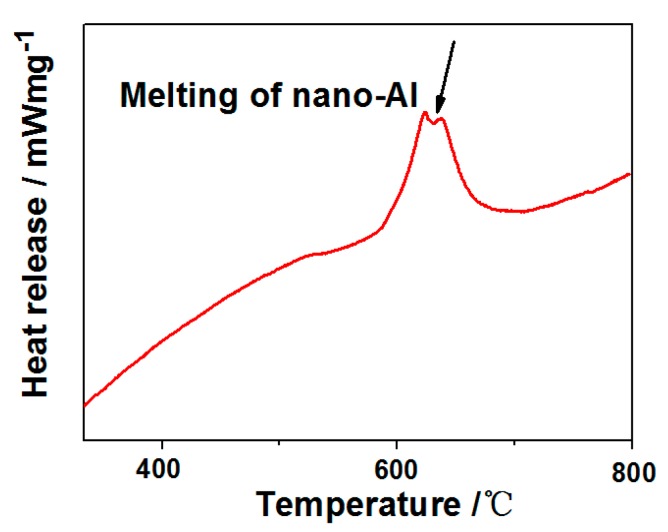
Thermal analysis results—the differential scanning calorimetry (DSC) curves of the SAFFs.

In order to analyze the heat-release performance of the SAFFs, all samples were characterized by the DSC technique. Generally speaking, the output of the heat is essential to the energetic materials or other kinds of explosive materials. In this special energetic system, energy release from the SAFFs was due to the process shown in Equation (1), and the corresponding specific exothermic process is recorded in Figure 4. Clearly, there is a sharp exothermic peak at ca. 600 °C, due to the strong chemical reaction between the nano-Al and the Fe_2_O_3_, in the composite films. There was a small endothermic peak at ca. 600 °C, resulting from the melting process of the nano-Al [37]. The total heat-release was up to 2.67 kJ/g, fitted by the DSC assistant software, which provided the thermal source theoretical foundation for the various potential applications of the SAFFs. 

### 3.4. Stability Analysis

**Figure 5:**
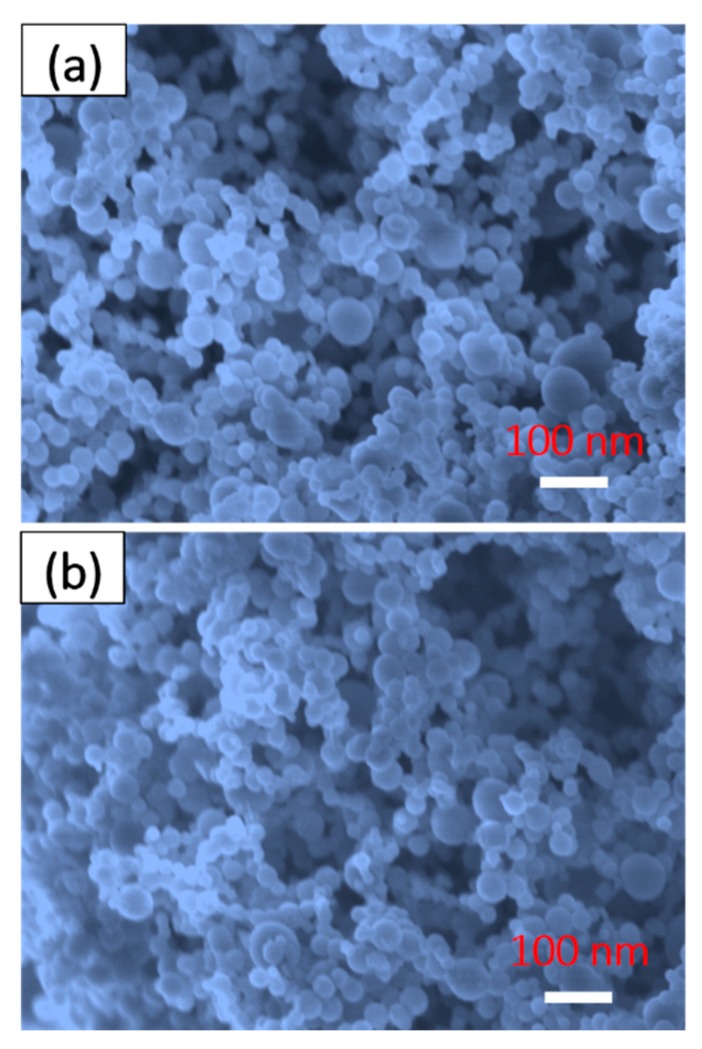
The typical FESEM images of the SAFFs before (**a**) and after (**b**) the exposure test for the six months.

For practical purposes, the effect of the variable environment on the water-proof property of product were analyzed, in detail, by adjusting the exposure time and the humidity. By comparing with Figure 5a,b, it can be seen that after going through a long exposure period of half a year, the SAFFs were almost unchanged, with an even distribution in the nano-scale and vast fascinating porous structures.

**Figure 6:**
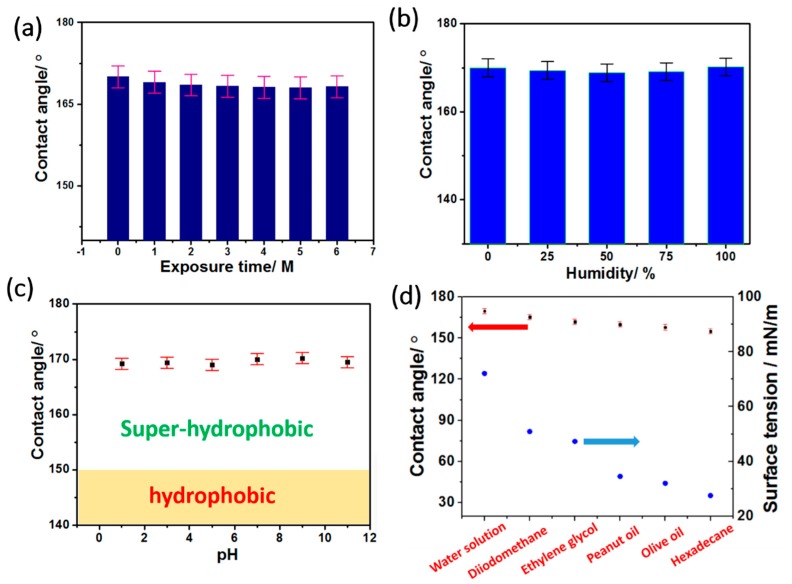
The contact angle as a function of exposure time (**a**), humidity (**b**), pH (**c**), and the different kinds of droplets (**d**).

The relationship of the contact angle and the exposure time is displayed in Figure 6a, where the contact angle of the target SAFFs was nearly 170° and barely got smaller with an increasing exposure time. Figure 6b displays the contact angle as a function of humidity, which was used to simulate a realistic environment. Clearly, there were few fluctuations on the contact angle of the samples, after six months of exposure, and the corresponding contact angle remained at a high level of 170°. Moreover, as the pH increased from 1 to 11, the contact angle also remained almost stable (Figure 6c), showing only a marginal effect of the pH, on the water-proof property of the product. What needed to be specially mentioned was that different droplets, with different surface tensions, including water, diiodomethane, ethylene glycol, peanut oil, olive oil, and hexadecane, were used to examine the practicability of the SAFFs. As shown in Figure 6d, the contact angle of the product decreased with the surface tension in the droplet. However, the contact angle of the SAFFs was still more than 150° (the “superhydrophobic” materials) even when the surface tension of the hexadecane was as low as 27.5 mN/m [30]. Thus, all results indicated the outstanding superhydrophobicity and stability of the product.

**Figure 7:**
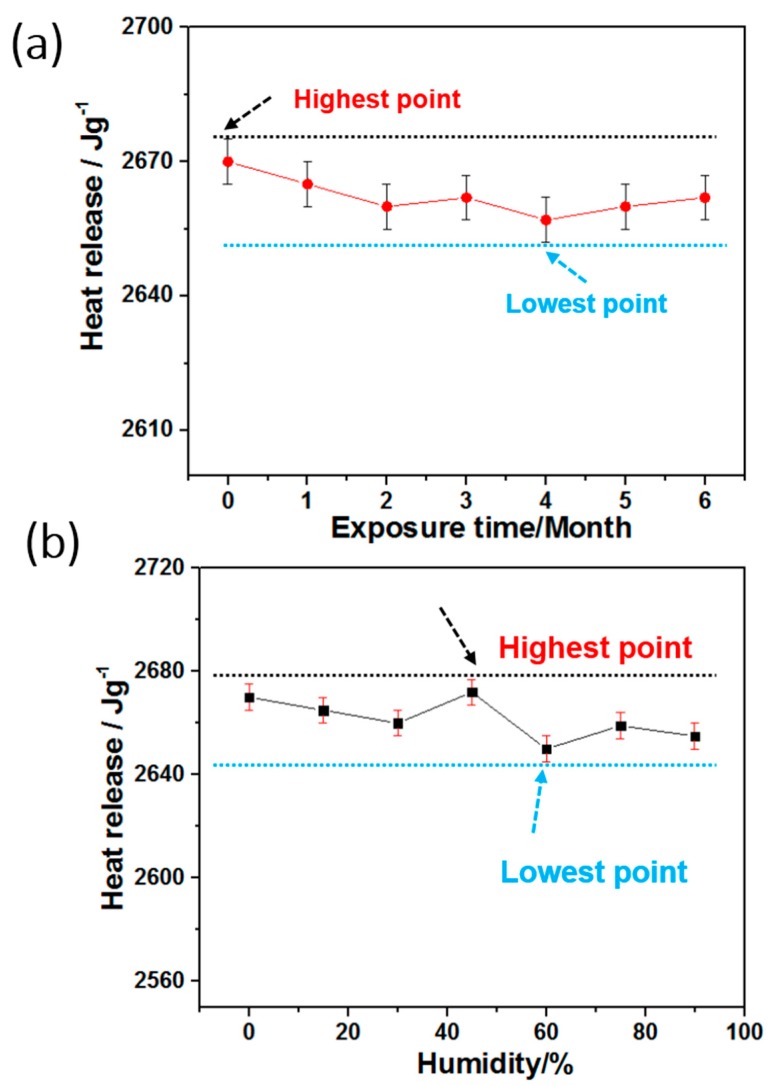
The relationship of the heat-release of products to (**a**) exposure time and (**b**) humidity.

Figure 7 shows the transformation law for the heat-release (Q) of the product, for various exposure times in the natural environment, and different humidity levels. As seen from Figure 7a, the internal chemical energy of SAFFs had a very small fluctuation, even after six months of exposure, and the fluctuation rate ($F_{r}$, calculated by the Equation (2)) was as low as 0.75%, showing a great heat stability.

(2) $$F_{r}=\frac{Q_{h}-Q_{l}}{Q_{i}}\times 100\%$$

where the $Q_{h}$, $Q_{l}$, and $Q_{i}$ represent the highest, lowest, and the initial heat-release value. 

In addition, the effect of the changeable humidity on the exothermic performance of the product was almost negligible, as shown in Figure 7b, and the corresponding $F_{r}$ was only 1.01%, which also indicated that the fabricated novel energetic materials, with ultra-long lifespan would have great potential applications in lots of domains.

## 4. Conclusions

In brief, SAFFs, with wide applications, have been fabricated by a facile one-step-process-controllable EPD technique. The resulting energetic product exhibited an outstanding superhydrophobicity, with a contact angle up to ca. 170°, and a great heat-release performance with Q up to 2.67 kJ/g, respectively. Moreover, the hydrophobic stability and exothermic stability of the SAFFs could be retained for at least six months, in changeable circumstances. Thus, this work provided a new perspective for designing novel energetic material with a high real-environment tolerance, for real industrial applications.

## Figures and Tables

**Table 1:** The results of the contact and the rolling angles of the SAFFs in five parallel experiments, under the same condition.

Parallel Experiments	Contact Angle/°	Rolling Angle/°
I	170.1 ± 1	1.0 ± 1
II	169.4 ± 1	0.9 ± 1
III	160.0 ± 1	0.9 ± 1
IV	168.9 ± 1	1.1 ± 1
V	169.1 ± 1	1.1 ± 1

## Supplementary Materials

The following are available online at http://www.mdpi.com/1996-1944/12/2/234/s1, Video S1: The droplet impacting process of the SAFFs, using red ink-dyed water, Video S2: The droplet impacting test of SAFFs fixed at a certain angle. The water droplets were sprayed on the surface and bounced-off quickly, suggesting the outstanding superhydrophobicity of the SAFFs.
