# Supplementary material for: Super-Efficient Synthesis of Mesh-like Superhydrophobic Nano-Aluminum/Iron (III) Oxide Energetic Films
Source: Materials (Basel). 2019 Jan 11;12(2):234. doi: 10.3390/ma12020234 (PMC6356539; doi:10.3390/ma12020234)
Supplement: Supplementary file 1 [file materials-12-00234-s001.zip › materials-403236-supplementary/supplementary.pptx]

## Slide 1
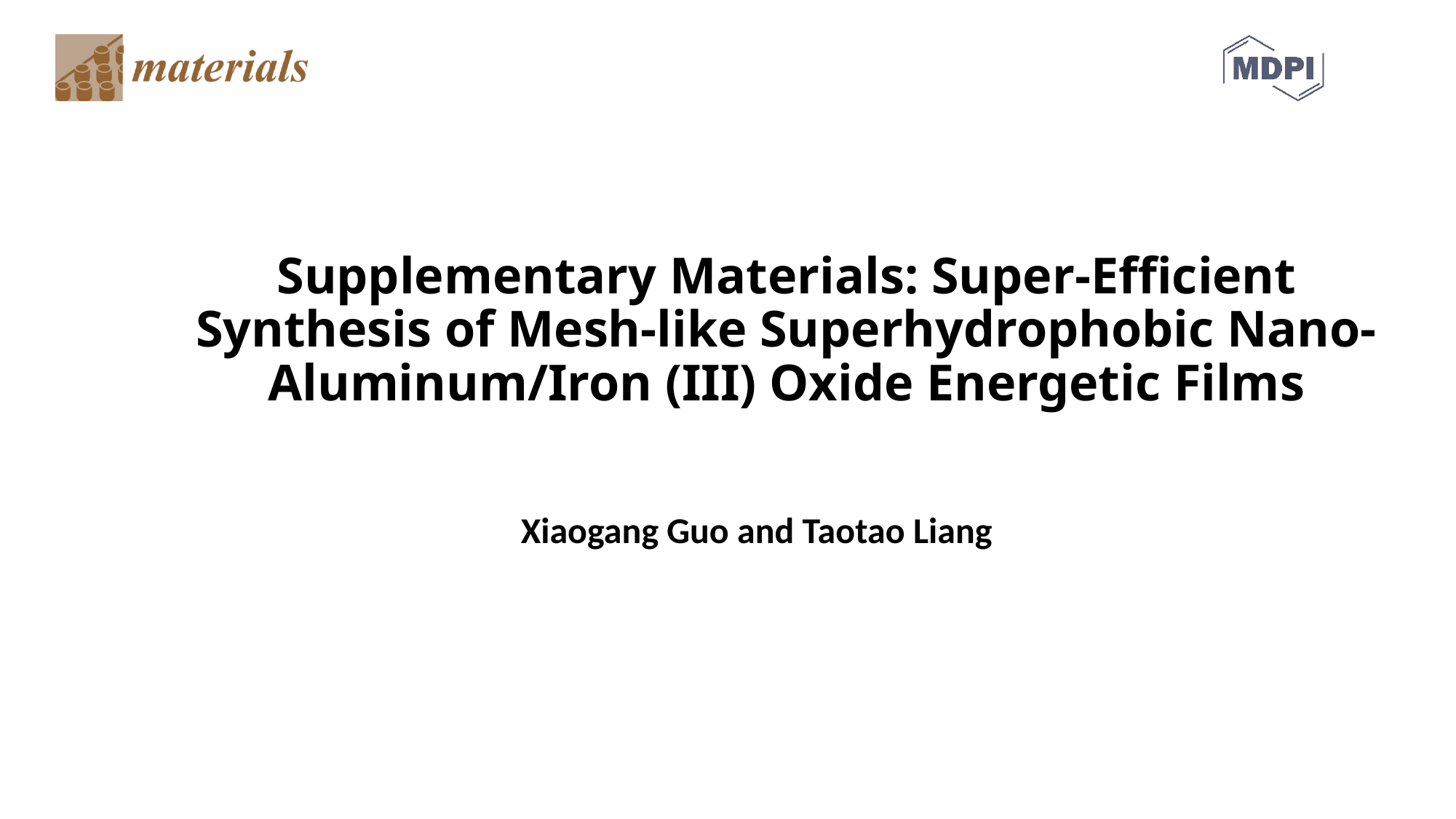

# Supplementary Materials: Super-Efficient Synthesis of Mesh-like Superhydrophobic Nano-Aluminum/Iron (III) Oxide Energetic Films
Xiaogang Guo and Taotao Liang

## Slide 2
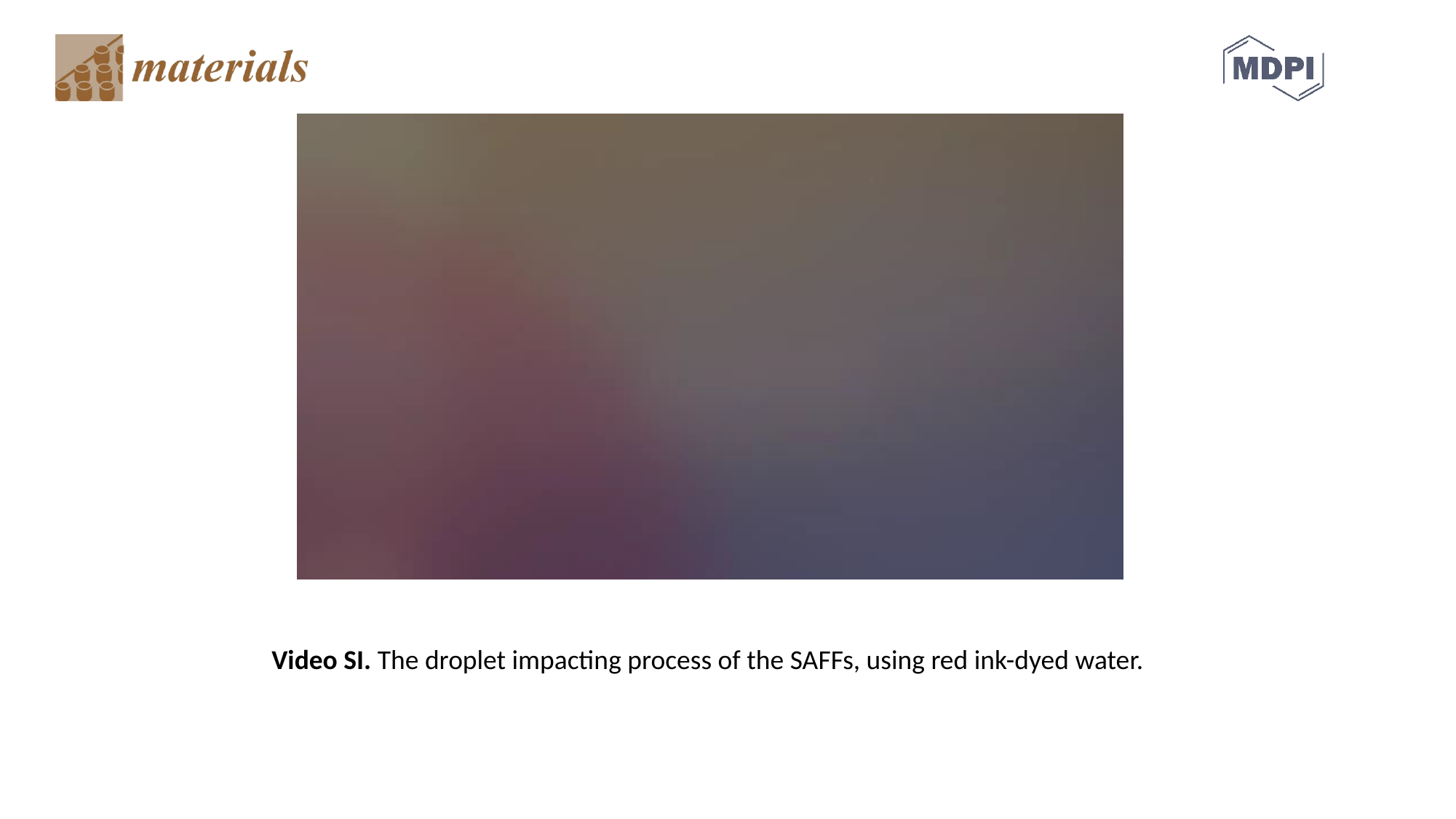

Video SI. The droplet impacting process of the SAFFs, using red ink-dyed water.

## Slide 3
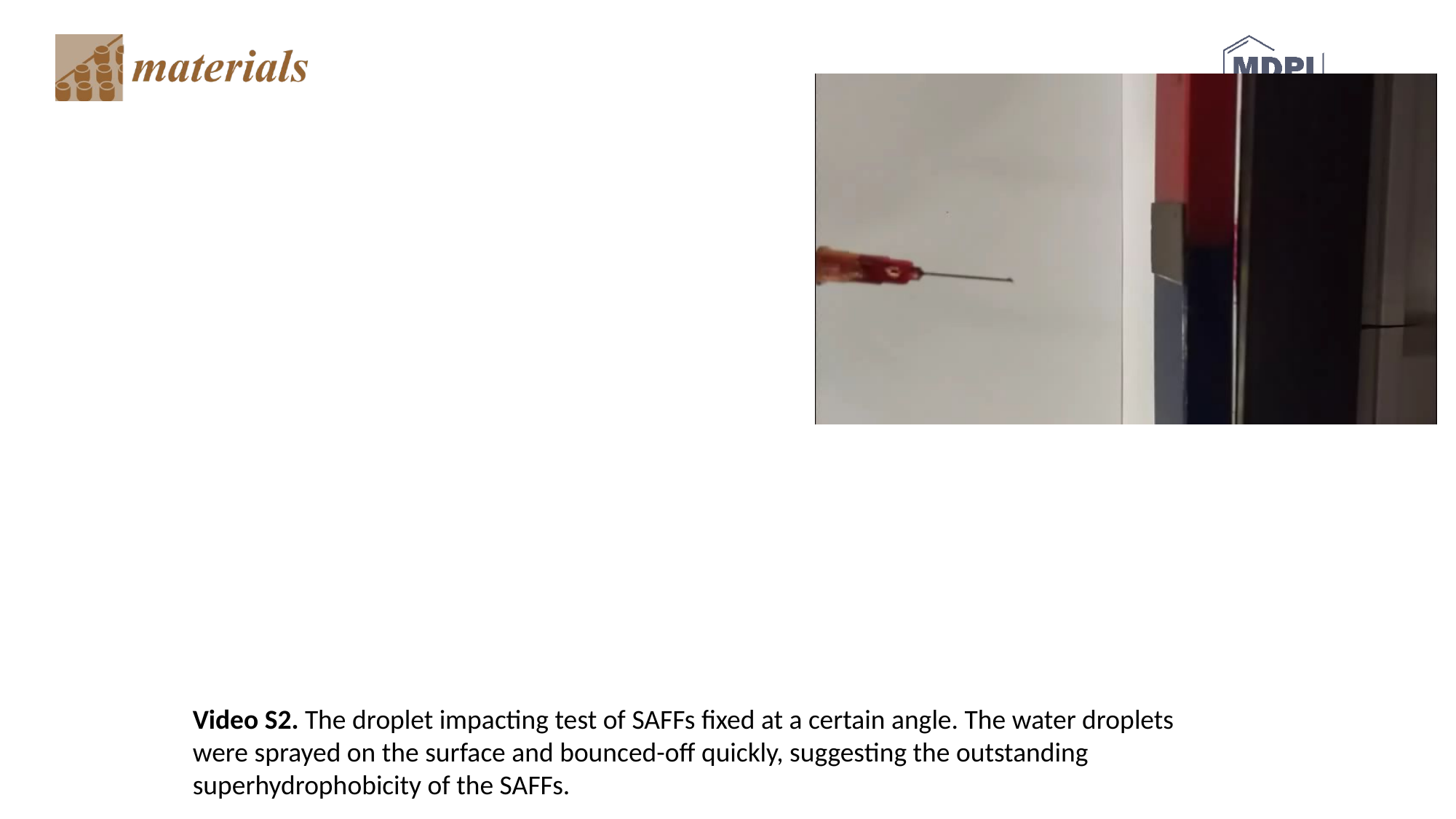

Video S2. The droplet impacting test of SAFFs fixed at a certain angle. The water droplets were sprayed on the surface and bounced-off quickly, suggesting the outstanding superhydrophobicity of the SAFFs.
